# Analysis of the Epigenome in Multiplex Pre-eclampsia Families Identifies *SORD*, *DGKI*, and *ICA1* as Novel Candidate Risk Genes

**DOI:** 10.3389/fgene.2019.00227

**Published:** 2019-03-19

**Authors:** Amir Ariff, Phillip E. Melton, Shaun P. Brennecke, Eric K. Moses

**Affiliations:** ^1^The Curtin UWA Centre for Genetic Origins of Health and Disease, Faculty of Health and Medical Sciences, Curtin University, The University of Western Australia, Perth, WA, Australia; ^2^School of Pharmacy and Biomedical Sciences, Faculty of Health Sciences, Curtin University, Perth, WA, Australia; ^3^Department of Maternal-Fetal Medicine Pregnancy Research Centre, The Royal Women’s Hospital, Melbourne, VIC, Australia; ^4^Department of Obstetrics and Gynaecology, The University of Melbourne, Melbourne, VIC, Australia; ^5^School of Biomedical Sciences, Faculty of Health and Medical Sciences, The University of Western Australia, Perth, WA, Australia

**Keywords:** epigenetics, whole-genome bisulfite sequencing (WGBS), pre-eclampsia, DGK, ICA, SORD, DNA methylation (CpG), differentially methylated region (DMR)

## Abstract

Pre-eclampsia is a serious heritable disorder that affects 5–8% of pregnancies worldwide. While classical genetic studies have identified several susceptibility genes they do not fully explain the heritability of pre-eclampsia. An additional contribution to risk can be quantified by examining the epigenome, in particular the methylome, which is a representation of interactions between environmental and genetic influences on the phenotype. Current array-based epigenetic studies only examine 2–5% of the methylome. Here, we used whole-genome bisulfite sequencing (WGBS) to determine the entire methylome of 13 individuals from two multiplex pre-eclampsia families, comprising one woman with eclampsia, six women with pre-eclampsia, four women with uncomplicated normotensive pregnancies and two male relatives. The analysis of WGBS profiles using two bioinformatics platforms, BSmooth and Bismark, revealed 18,909 differentially methylated CpGs and 4157 differentially methylated regions (DMRs) concordant in females. The methylation patterns support the involvement of previously reported candidate genes, including *COL4A1, SLC2A4, PER3, FLT1, GPI, LCT, DDAH1, TGFB3, DLX5*, and *LRP1B*. Statistical analysis of DMRs revealed three novel genes significantly correlated with pre-eclampsia: sorbitol dehydrogenase (*SORD*, *p* = 9.98 × 10^-6^), diacylglycerol kinase iota (*DGKI*, *p* = 2.52 × 10^-5^), and islet cell autoantigen 1 (*ICA1*, 7.54 × 10^-3^), demonstrating the potential of WGBS in families for elucidating the role of epigenome in pre-eclampsia and other complex diseases.

## Introduction

Pre-eclampsia (PE) is a pregnancy-related disorder clinically defined by hypertension and proteinuria after the 20th week of gestation. Several studies of different cohorts report a prevalence of up to 8% of pregnant women worldwide (Saftlas et al., 1990; Sibai et al., 1995; Duley, 2009; Ghulmiyyah and Sibai, 2012; Ananth et al., 2013; Rezende et al., 2016), resulting in the disorder being a major cause of maternal and foetal morbidity and mortality. The genetic component attributable to phenotypic variation in this disorder, heritability, ranges from 30 to 60% (Moses et al., 2000; Salonen Ros et al., 2000; Cnattingius et al., 2004; Nilsson et al., 2004; Carr et al., 2005; Johnson et al., 2007; Thomsen et al., 2015), and twin studies suggest a heritability component of 24% (Thornton and Macdonald, 1999). Classically, several candidate genes were interrogated for association with PE, including the collagen superfamily genes, such as collagen α1(I) chain (*COL1A1*) and collagen α2(IV) chain (*COL4A2*) (Nakayama et al., 2004; Goddard et al., 2007; Enquobahrie et al., 2011; He et al., 2015); interleukins, such as IL-1α (*IL1A*) (Goddard et al., 2007); haemodynamic genes related to the renin-angiotensin system, such as *AGT*, *REN*, *ACE*, *AGTR1*, and *AGTR2* (Lévesque et al., 2004; Plummer et al., 2004; Roberts et al., 2004; GOPEC Consortium, 2005; Buurma et al., 2013); lymphotoxin-α (*LTA*) (Nakayama et al., 2004); and clotting factors, such as factor V (Buurma et al., 2013) and von Willebrand factor (*VWF*) (Nakayama et al., 2004). However, replication of these results have proven difficult and inconsistent, with some studies even finding no evidence for such associations (Lachmeijer et al., 2001b; Livingston et al., 2001; Serin et al., 2002; Nakayama et al., 2004; Goddard et al., 2007; Bombell and McGuire, 2008). This suggested that there remained genes and genomic sequences whose roles in PE haven’t been identified, a concept which was then explored via genome wide association studies (GWAS), resulting in the identification of susceptibility genes such as, *STOX1* (van Dijk and Oudejans, 2011; Ducat et al., 2016; Vaiman and Miralles, 2016) and *ACVR2A* (Moses et al., 2006; Fitzpatrick et al., 2009; Roten et al., 2009; van Dijk and Oudejans, 2011; Ferreira et al., 2015), as well as various quantitative trait loci (QTLs) on chromosomes 2, 4, 5, 7, 9, 10, and 13 (Arngrímsson et al., 1997, 1999; Harrison et al., 1997; Moses et al., 2000, 2006; Lachmeijer et al., 2001a; Laivuori et al., 2003; van Dijk et al., 2005; Johnson et al., 2007). Even so, these results did not fully explain the heritability of the disorder, further suggesting that simple genetic inheritance does not sufficiently explain the situation, and the high discordance of PE phenotype in twins (Treloar et al., 2001) implies the involvement of more than just a genetic component, leading us to study epigenetics as a possible bridge between genetic and environmental factors.

Epigenetics was first linked to human diseases when differential methylation patterns were observed in diseased tissue from patients with colorectal cancer compared to normal tissue (Feinberg and Vogelstein, 1983). Since then, epigenetics has been used to explain a component of various diseases, particularly different types of cancers, and provides a discrete explanation to disease variations in genetically identical twins (Relton and Davey Smith, 2010; Sharma et al., 2014; van Dijk et al., 2014; Dolan et al., 2015; Loddo and Romano, 2015; Maltby et al., 2015; Zeybel et al., 2015; Hannon et al., 2016; Ligthart et al., 2016; Watson et al., 2016).

The epigenetics of PE have previously been studied with regards to global and gene-specific methylation patterns (Chelbi et al., 2007; Kulkarni et al., 2010; Yuen et al., 2010). Differential DNA methylation patterns have been implicated and associated with PE in various studies, identifying genes such *as POMC, AGT, CALCA, DDAH1, TGFB,* and *DLX5* (Blair et al., 2013; Anton et al., 2014; Chu et al., 2014; Liu et al., 2014; Martin et al., 2015; White et al., 2016; Xuan et al., 2016; Zadora et al., 2017). However, these studies employed directed sequencing methods via the use of microarray chips and transcriptomic analyses, limiting the coverage of sequencing and thus the reportable resultant methylated regions, and there seems to be no overlap in findings of the different studies. Additionally, these studies predominantly assess DNA methylation patterns collected from placental biopsies, which is known to have a skewed, hypomethylated profile compared to both mother and child (Ehrlich et al., 1982; Fuke et al., 2004; Price et al., 2012; Schroeder et al., 2013; Fogarty et al., 2015; Martin et al., 2015; Robinson and Price, 2015; Bianco-Miotto et al., 2016). Although it seems logical to target methylation patterns in such a tissue of interest, we believe that methylation profiles from blood samples may provide a more holistic overview of the epigenetic environment of both mother and child, as well as being unbiased according to specific tissue type (Ehrlich et al., 1982; Price et al., 2012; Schroeder et al., 2013). Additionally, results of analysis from blood methylation profiles is more pragmatic for the development of PE detection and diagnostic tools.

Array-based sequencing only captures approximately 5% of the whole-genome, whereas whole-genome bisulfite sequencing (WGBS) expands upon this, allowing the capture and analysis of methylation patterns across whole-genomes. Additionally, array-based sequencing generally only analyses methylation in a CpG context, whereas in WGBS, three categories of methylation contexts that comprise the majority of known methylation sites are analysed: those in CpG sites, CHH sites, and CHG sites, allowing a more comprehensive analysis of the methylome. As this is a relatively new technology, research in the field is in its early stages. A preliminary WGBS study on two twin pairs revealed 12 candidate differentially methylated regions (DMRs) associated with hypertensive pregnancy disorders (Oudejans et al., 2016), and a recent WGBS study on placental tissue has reported that DMRs associated with PE are involved in the following cellular pathways: cell adhesion, wingless type MMTV Integration Site family member 2 (Wnt) signalling pathway, and regulation of transcription (Yeung et al., 2016).

Herein we have utilised a WGBS approach in multiplex families to identify DMRs associated with PE; using a conservative bioinformatics approach we assessed methylation patterns in 13 individuals across two multiplex families and identified DMRs associated with three genes that are statistically correlated with PE: sorbitol dehydrogenase (*SORD*), diacylglycerol kinase iota (*DGKI*), and islet cell autoantigen 1 (*ICA1*), which are novel and biologically plausible candidate genes. To our knowledge, this is the first study of multiplex families utilising WGBS of maternal blood to elucidate methylation patterns in relation with PE.

## Materials and Methods

### Subjects – Pre-eclampsia Families

The 13 individuals in the two multiplex pre-eclampsia families investigated in this study were of European ancestry and are a subset of the total family based cohort of 74 families that we have described previously (Moses et al., 2000, 2006; Johnson et al., 2007, 2012, 2013; Fitzpatrick et al., 2009). The 13 individuals included 1 woman with eclampsia, 6 women with severe pre-eclampsia, 4 women who had normotensive pregnancies, and 2 male relatives (Supplementary Figure S1). This study was approved by the University of Western Australia Human Research Ethics Office under project number RA/4/1/5759 and all methods were performed in accordance with the relevant guidelines and regulations. All participants provided written informed consent for sample collection and use in this study.

### Pre-eclampsia Diagnosis

Pre-eclampsia diagnosis was conducted by qualified clinicians using criteria set by the Australasian Society for the Study of Hypertension in Pregnancy (Brown et al., 1993; Brown et al., 2000) and the Society of Obstetric Medicine of Australia and New Zealand for the management of hypertensive diseases of pregnancy (Lowe et al., 2009). Pre-eclampsia was defined in pregnant women if they were previously normotensive and if they had on at least two occasions six or more hours apart, after 20 weeks of gestation (i) a rise in systolic blood pressure (SBP) of at least 25 mmHg and/or a rise from baseline diastolic blood pressure (DBP) of at least 15 mmHg, or (ii) SBP ≥ 140 mmHg and/or DBP ≥ 90 mmHg. In addition, significant new onset proteinuria levels were either ≥0.3 g/l in a 24 h specimen, at least a “2+” proteinuria dipstick reading from a random urine collection or a spot protein/creatinine ratio 30 mg/mmol (0.3 mg/mg). Women with pre-eclampsia who had experienced convulsions or unconsciousness in their perinatal period were classified as having eclampsia. Women with pre-existing hypertension or other medical conditions associated with pre-eclampsia (e.g., renal disease, diabetes, twin pregnancies, or foetal abnormalities) were excluded.

### Whole-Genome Bisulfite Sequencing (WGBS)

Genomic DNA samples were sequenced at an average depth of 30X using the Illumina HiSeq X Ten WGBS kit (CA, United States), conducted by Macrogen (Seoul, South Korea). Sample preparation, library construction, and sequencing was performed according to the protocols prescribed by Macrogen. Base calling on the generated raw reads was performed through the integrated primary analysis software, RTA 2 (Real Time Analysis 2). The base called binary file was converted into FASTQ format using Illumina package bcl2fastq v2.15.0, and resulting reads were used as the input for further bioinformatics analysis. Raw data statistics are listed in Supplementary Table S1.

### Bioinformatics Analysis

Initial quality control on raw sequences was conducted using Trim Galore v0.4.3^1^, where sequences were filtered against reads with biassed methylation patterns, had a Phred score lower than 20, and/or contained standard Illumina adapters. Read quality and metrics were subsequently visualised and curated using FastQC v0.11.3^2^. Filtered read statistics are listed in Supplementary Table S2.

Remaining reads were indexed and aligned with Bismark v0.17.0 (Krueger and Andrews, 2011) and Bowtie2 v2.2.6 (Langmead and Salzberg, 2012). Alignment was performed against the human reference genome build hg19, only including chomosomes 1–22, X, and Y; Bismark v0.17.0 was paired with Samtools v0.1.19 (Li et al., 2009) to deduplicate reads and extract methylation data. Only methylation sites in the context of CpG dinucleotides, CHG trinucleotides, and CHG trinucleotides (where H denotes any nucleotide aside from G) were retained for Bismark analysis.

Subsequent methylation analysis was performed using two platforms: (1) BSmooth (Hansen et al., 2012) and (2) RnBeads (Assenov et al., 2014). Methylated sites were compared between cases and controls, while accounting for the two different families as a co-variate. Using BSmooth, DMRs were defined when containing at least 3 CpGs, were present in at least two samples, had a cut-off t-statistic of more than 4.6 or less than -4.6, and had a t-distribution group mean difference between case and control of larger or equal to 0.1. While analysing sequences with RnBeads, greedycut filtering was disabled due to the large dataset. Import bed style was set to “bismarkCov,” single nucleotide polymorphism (SNP)-enriched sites were removed, and differentially methylated sites in a CpG context were analysed according to the following categories: (1) CpG islands, (2) genes, (3) promoter regions, and (4) all sites combined. Results from analysis on both platforms were compared and overlapping methylated sites and DMRs reported herein. Methylation extents are reported as β-values as defined by RnBeads. DMCpGs and DMRs were considered statistically significantly differentiated if the FRD-adjusted β-values were less than 0.05.

Differentially methylated regions associated with a cohort of 80 candidate genes, identified from previous studies (Supplementary Table S3), were extracted from this data, as well as DMRs associated with three previously identified QTLs: 2q22 (chr2:132900000–166500000) (Moses et al., 2006), 5q (chr5:76057719–119149278) (Johnson et al., 2007), and 13q (chr13:103876397–112895563) (Johnson et al., 2007). DMRs annotated by both bioinformatics platforms were defined as being associated with genes if they were located in the gene or 500 kbp up- or downstream of the gene.

## Results

### Whole-Genome Bisulfite Sequencing (WGBS)

Whole-Genome Bisulfite Sequencing was conducted on 13 individuals across two multiplex PE families using Illumina paired-end technology at an average depth of 30X, with an average read length of 150 basepairs (bp). At least 90% of the reads were of Phred score 20 or higher, and more than 50% of these reads were retained after removing duplicate and unaligned reads. Approximately 7.06 × 10^9^ methylated sites were identified per genome. On average, 4.50 × 10^8^ CpG, 1.73 × 10^9^ CHG, and 4.88 × 10^9^ CHH putative methylation sites were identified per genome, with methylation rates heavily favouring the CpG sites average methylation rates of 77.84, 2.95, and 1.94%, respectively, Supplementary Table S2. There is an apparent skew in the number of methylated sites per chromosome, in particular, hypermethylation of chr17 and chr19 (χ^2^ = 2640 and 2885, respectively).

### Differentially Methylated Cytosine Residues Support Previous Findings in Candidate Genes

RnBeads identified 27,840,614 unique differentially methylated CpG sites (DMCpGs) across all female samples, of which 27,347 were associated with CpG islands, 53,938 with annotated genes, and 57,356 with promoter regions; Bismark identified 3,346 DMRs (Supplementary Figure S2). Potentially disruptive CpG sites that overlapped with common SNPs were removed from RnBeads analysis, resulting in the removal of 362,168 sites. No significant bias was found when gender and PE status were considered as covariates, though there were significant differences depending on family, confirming that inherited epigenetic patterns are a major confounder for such analyses. Methylation data from the mitochondrial chromosome was excluded due to its small size. A concordance of these sites and regions between both bioinformatics platforms resulted in a remaining 3346 DMRs containing 15,156 DMCpGs. When only female samples were analysed, this number was increased to 4157 DMRs containing 18,909 DMCpGs. Subsequent analysis was conducted on the DMRs and DMCpGs extracted from comparing female samples. Three hundred and two DMCpGs were associated with QTLs previously identified by linkage mapping in these Australian and New Zealand families at 2q22 (Moses et al., 2006), 5q (Johnson et al., 2007), and 13q (Johnson et al., 2007), comprising of 29, 35, and 7 DMRs, respectively. Additionally, the following candidate genes were associated with identified DMRs: COL4A1, SLC2A4, PER3, FLT1, GPI, LCT, DDAH1, TGFB3, DLX5, and LRP1B. The DMRs were filtered by number of DMCpGs (minimum count of 10), and density of DMCpGs, and the top hits were curated. Although these DMRs are associated with previous data, in this cohort, the FDR-corrected *p*-values did not meet significant thresholds (<0.05) to be further analysed. No apparent pattern was revealed, however, these DMRs were generally associated with protein kinases, ionic channels, G-protein signalling, and low-density lipoprotein receptors. Interestingly, pairing the data with whole-genome sequence analysis revealed that although these genes harboured potentially influential SNPs related to PE, there was no statistical correlation between these SNPs and PE (data not shown).

**FIGURE 1:**
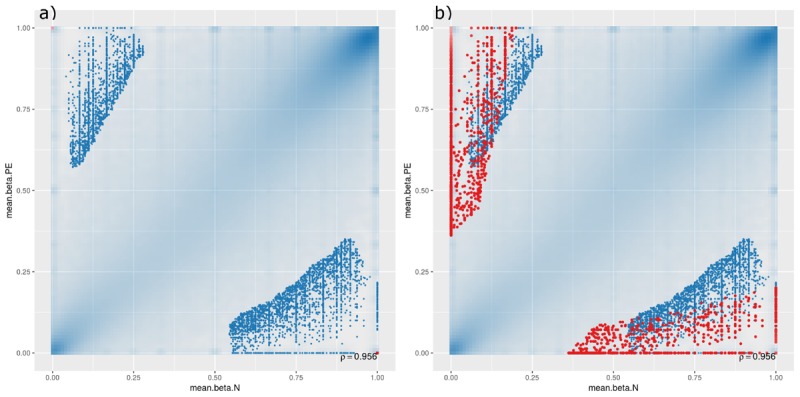
Site-level differentially methylated cytosine residues (DMCpGs) comparing pre-eclamptic to non-pre-eclamptic samples. Site-level differentially methylated cytosine residues in CpG context (DMCpGs) comparing mean β-values between cases of pre-eclamptic samples (PE, *y*-axis) and controls of non-PE samples (N, *x*-axis). All data points with an unadjusted *p*-value of less than 0.05 are represented as blue dots in both panels. Additionally, red dots represent the top ranking 1000 hits in (panel **a**) (unique to either PE or non-PE samples), and top ranking 10,000 hits in (panel **b**).

A scatterplot was generated to compare DMCpGs between PE and normal samples at the site level (Figure 1), which showed that, though there are significant DMCpGs between the two groups, the top 1000 significant hits had mean β-values in both PE and non-PE cohorts larger than 0.5 (Figure 1a), and hits with β-values smaller than 0.5 in either cohort only began to appear when the top 10,000 or more hits were accounted for (Figure 1b). As such, two groups of DMCpGs were identified, those being hypermethylated in the PE cohort (top left data points in Figure 1), and those being hypomethylated in the PE cohort (bottom right data points in Figure 1). None of these hits were considered significant after adjusting for false detection rates (FDR) at a *p*-value of 0.05. This suggests that, in this study, individual DMCpGs may not account for differences between PE and normal groups.

**FIGURE 2:**
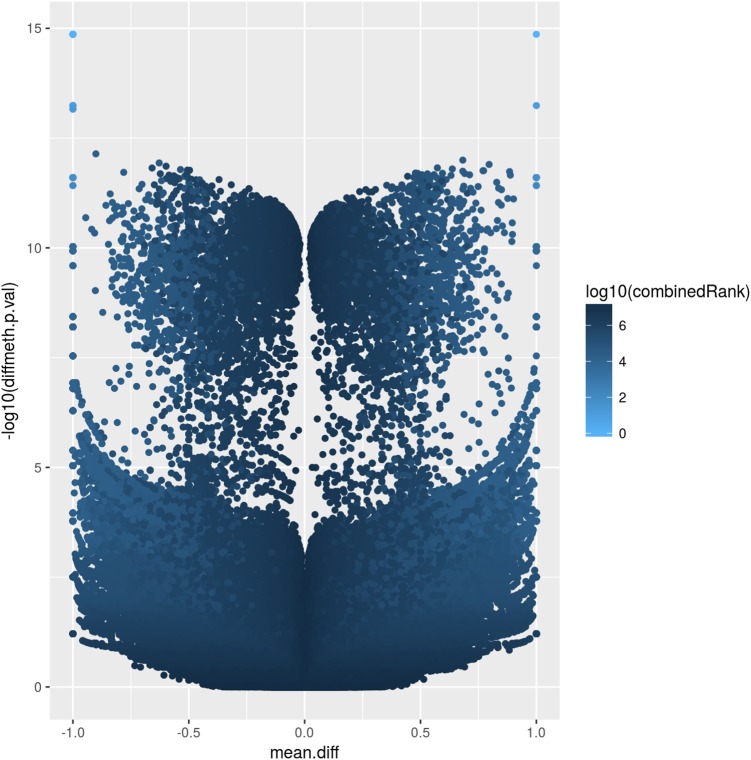
Volcano plot of site-level differentially methylated cytosine residues in CpG context (DMCpGs). The *y*-axis denotes *p*-values of DMCpGs with a –log_10_ transformation, whereas the *x*-axis denotes a mean fold difference between cases of pre-eclampsia (PE) against non-PE controls. Data points with *x*-values lower than 0 represent relative hypomethylation, and those with *x*-values larger than 0 represent relative hypermethylation. The intensity of each data point correlates with a combined rank score attributed by RnBeads as per the scale bar on the right.

The corresponding volcano plot (Figure 2) supports this observation, as individual DMCpGs did not cluster according to a typical distribution. Instead, it suggests that amongst the significantly different DMCpGs (adjusted β-values < 0.05), there is a subset of sites that are highly correlated with PE (both by hypo- and hypermethylation), which may be more pragmatically analysed as DMRs. DMCpGs with less relative hypo- and hypermethylation ratios were correlated with higher combined rank scores and *vice versa*, suggesting that the PE phenotype cannot be explained by individual DMCpGs, but as a cumulation of DMCpGs with small-effect sizes.

### Three Novel Genes, SORD, DGKI, and ICA1 Are Associated With DMRs in PE Cases

**FIGURE 3:**
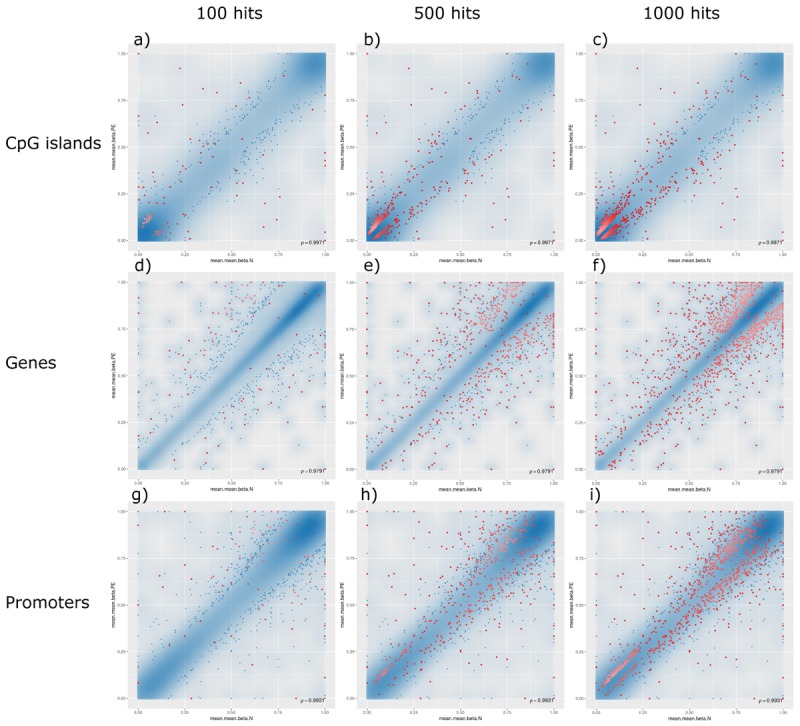
Methylation levels comparing pre-eclamptic to non-pre-eclamptic samples. Mean β-values of differentially methylated regions (DMRs) in pre-eclamptic samples (PE, *y*-axis) against non-PE samples (N, *x*-axis). In all panels, data points with an unadjusted *p*-value of less than 0.05 are represented as blue dots. Each row, respectively, represents data for DMRs associated with CpG islands (panels **a–c**), genes (panels **d–f**), and promoters (panels **g–i**). Each column, respectively, represents top hits with cutoff thresholds of top ranking 100 hits (panels **a,d,g**), top 500 ranking hits (panels **b,e,h**), and top 1000 ranking hits (panels **c,f,i**), where each data point meeting these criteria are represented as red dots.

The analysis of such DMRs revealed that, although there were top hits associated with PE with significant β-values, no CpG islands or promoter-associated DMRs were individually associated with PE after FDR adjustment at a *p*-value of 0.05; however, some potential gene-associated DMRs did meet the FDR requirement and remain statistically significantly associated with PE. Analysing the non-statistically significant hits, the following trends were observed: (1) generally, DMRs were distributed along the linear line correlating methylation patterns in both PE and non-PE cohorts; (2) DMRs associated with CpG islands were generally hypomethylated in both cohorts, and increasing the threshold for reported DMRs from top 100 (Figure 3, panel a), to top 500 (Figure 3, panel b), to top 1000 (Figure 3, panel c) increasingly populated the hypomethylated data points (bottom left of figures); (3) the converse is observed for DMRs associated with genes, where DMRs are generally similarly methylated in both cohorts, but increasing the threshold from reported DMRs increasingly populates hypermethylated data points (top right of figures, Figure 3 panels d–f); and (4) DMRs associated with promoter regions do not show any over association between methylation patterns and PE status (Figure 3, panels g–i).

Volcano plots for DMCpGs, as well as all DMR categories (genes, promoters, and CpG islands) relating combined *p*-values and fold increase in differential expression showed no significant hits which were both differentially expressed and had high combined *p*-values, further supporting the scatterplot data described (Figure 3). Adjusting for FDR values with *p* < 0.05 resulted in 4 DMRs associated with genes that met the criteria: chr15:45365690–45366246 (sorbitol dehydrogenase, *SORD*, *p*-value = 9.98 × 10^-6^), chr7:137982833–137983229 (diacylglycerol kinase iota, *DGKI*, *p*-value = 2.52 × 10^-5^), chr7:8194566–8194652 (islet cell autoantigen 1, *ICA1*, *p*-value = 7.54 × 10^-3^), and chr4:83095700–83095806 (pseudogene U6, *RNU6-499P*, *p*-value = 0.0268). The DMR associated with *ICA1* is also associated with the genes cAMP response element-binding protein 3-like 2 (*CREB3L2*) and aldo-keto reductase family 1 (*AKR1D1*), and are discussed below as being associated with *ICA1*. We examined the genes whose DMRs are statistically associated with PE in the studied families, after correction for FDR (Table 1). The pseudogene, RNU6-499P, was found to be statistically significantly associated both with PE and family (the latter acting as a negative control). As such, we excluded it from analysis, and examine the remaining genes, *SORD*, *DGKI*, and *ICA1*.

**Table 1:** Methylation data for novel identified genes in this study, SORD, DGKI, ICA1, and RNU6-499P.

ENSEMBL ID	Chromosome	Start^a^	End^a^	Symbol	Mean.N^b^	Mean.PE^b^	Mean.diff^b^	Comb.p.val^c^	Comb.p.adj.fdr^c^	CombinedRank^c^
ENSG00000259352	chr15	45365690	45366246	SORD	1	0.673809524	0.32619	1.86E-10	9.98E-06	888
ENSG00000213238	chr7	137982833	137983229	DGKI	1	0.912643994	0.087356	9.39E-10	2.52E-05	7238
ENSG00000265212	chr7	8194566	8194652	ICA1	0	1	-1	5.10E-07	0.007543012	3
ENSG00000202485	chr4	83095700	83095806	RNU6-499P	0.6667	1	-0.33333	2.49E-06	0.026763498	843


^*a*^The columns “Start” and “End” indicate the starting and ending chromosomal positions of the differentially methylated regions identified, as defined in the UCSC genome build hg19. ^*b*^The mean methylation coefficient β for each category of normotensive (mean.N), pre-eclamptic (mean.PE), and difference between these values (mean.diff), and the as defined by the software, Bismark. ^*c*^The statistical significance of difference between methylation extent of normotensive and pre-eclamptic samples calculated as a combined unadjusted *p*-value (comb.p.val) and a combined false-detection-rate-adjusted *p*-value (comb.p.adj.fdr).

## Discussion

To begin assessing the methylation data described, we compared the number of methylated sites with that in the literature and found it comparable to previous estimates of 28 million CpGs (Stevens et al., 2013), with an average CpG read coverage of approximately 24x. Additionally, we found that methylation rates were approximately 80% in CpG context, a finding comparable to that of methylation rates in adult human brain tissue (Guo et al., 2013), placental tissue and cord blood (Finer et al., 2015), general methylation rates across different human tissue cell types (Ehrlich et al., 1982), as well as general methylation rates in vertebrates (Jabbari and Bernardi, 2004). Other studies suggest that placental methylation rates may be lower than this, however, these data derive from microarray analyses, and may not fully reflect whole-genome methylation rates (Kulkarni et al., 2010; Yuen et al., 2011; Chu et al., 2014; Finer et al., 2015).

Analysis conducted while including male samples did not produce any significant results, whereas there were some gene-associated DMRs correlated with PE when only analysing female samples. Additionally, when accounting for gender, methylation patterns were as comparably associated with PE status as they were with the family analysed: no significant results of *p*-values below 0.01 when testing PE status against methylation patterns in the categories for CpG islands, genes, promoter regions, and individual sites, using eight principal components via Wilcoxon tests. Of the eight principal components, the most influential component, PC1, was found via Wilcoxon test to be significantly associated with family status for each methylation pattern category: CpG islands (*p* = 8.7 × 10^-3^), genes (*p* = 4.3 × 10^-3^), promoter regions (*p* = 4.3 × 10^-3^), and individual sites (*p* = 4.3 × 10^-3^). This may suggest that methylation analysis is only relevant in the setting of family cohorts, and may not be useful as a general predictive tool for PE in isolation. Additionally, although we could not correlate these methylome analysis results with whole-genome sequencing data on the same individuals, it is possible that the lack of statistical significance in this comparison is attributable to the absence of methylation-state analysis, however, we are unable to confirm this hypothesis without a larger cohort of putative genes to analyse.

The atypical volcano plot relating methylation rates of DMCpGs and *p*-values in PE and non-PE samples (Figure 2) indicated that there were two roughly symmetrical distributions of hypo- and hypermethylated DMCpGs, which were replicated when comparing between the two families. This may suggest that the methylation patterns are not strongly influenced by PE, and may instead be explained by normal individual variation. Such a hypothesis may only be confirmed by increasing the number of samples analysed, which may provide insight into family based methylation patterns, and help determine if PE can be predicted by methylation patterns on a family-to-family basis, rather than on an individual basis. We hypothesise that in large families, especially with complex hierarchical structures, we are better able to use methylation patterns as a predictor of PE, as such data would smoothen the distribution of DMCpGs, and allow the analysis of methylation patterns controlling for individual variation we observe herein.

Additionally, we attempted to explain the two groups of hypo- and hypermethylated DMCpGs as being of small effect-size (data points with less than 10^-5^ fold *p*-value between PE and non-PE in Figure 2), and large effect-size (more than 10^-5^ fold *p*-value ratio between PE and non-PE in Figure 2). As a complex disorder, it would follow that PE is influenced by the culmination of methylation patterns of DMRs containing a mixture of both these categories.

Sorbitol dehydrogenase mutations have been shown to cause secondary complications of diabetes (Carr et al., 2004), which are known predisposing or confounding factors to PE. Indeed, PE can be considered a complex cardiovascular disease, and its relationship with diabetes and other cardiovascular diseases is established in the literature (Baumfeld et al., 2015; Salzer et al., 2015; Wright et al., 2015; Kalafat and Thilaganathan, 2017; Wu et al., 2017; Vestgaard et al., 2018). At the molecular level, *SORD*, alongside aldose reductase, has been shown to mediate the polyol pathway, directly correlated with oxidative stress in the context of diabetic cardiovascular complications (Jay et al., 2006), which is a possible mechanism of action of PE symptoms.

In the same light, we evaluated the other genes in relation to cardiovascular diseases and diabetes. *DGKI*, as with other diacylglycerol products play a role in the tonal contraction of vascular smooth muscle, which has a role in hypertension (Ohanian and Ohanian, 2001; Choi et al., 2009). Islet cell autoantigens, including *ICA1*, have been associated with the development of type I diabetes mellitus, and there is defined structural and pathway evidence linking the gene product to disease (Arvan et al., 2012). Though there is no direct evidence of the involvement of *DGKI* or *ICA1* with PE, the pathways involved are plausible biological candidates for generalised modulation mechanisms whose dysregulation may contribute to PE, especially when occurring in concordance with other PE risk factors. *CREB3L2* is a transcription factor that is expressed in most tissues, with strongest expression in placenta, amongst other organs; whereas *AKR1D1* reduces cholesterols, including progesterone and testosterone, to 5-β-reduced metabolites, and is associated with neonatal cholestasis, a metabolic disease of infants that presents as jaundice.

Traditional genome-wide association studies have published thousands of common variants whose allele frequencies have been statistically correlated with various phenotypes. However, the majority of these variants have not been able to predict or biologically explain the phenotypes, be they individually or collectively. Although a criticism of GWAS is beyond this study, it is apparent that more complex analyses are required to understand the underlying genetics and genomics of complex diseases. In this study with only a small sample size we present evidence that genome-wide analysis of DNA methylation in the human methylome adds a level of complexity unto traditional sequencing data that may potentially explain phenotypes not yet elucidated. Genetic variation alone cannot thoroughly explain the phenotypes of complex diseases, and the influence of environmental factors has only been recently explored; as such, epigenetics, being directly modulated by environmental influences, provides a useful preliminary measure to quantitatively bridge genetic and environmental data.

It is worth mentioning that the statistical association of DMRs with PE herein does not imply causality, or even consequence. As with all complex diseases, the polygenic influence on phenotype cannot simply be ascertained via a singular sequencing analysis; indeed, it is shown that methylation patterns may be influenced by complications of pregnancy. One may endeavour to utilise the data from this pilot study to confirm the presence and role of differentially methylated patterns with targeted methylation sequencing in longitudinal studies.

In this study the use of family structures has allowed for normalisation of individual and familial variation in methylation patterns, allowing for a more accurate identification of DMRs associated with PE. As such, we believe that continued interrogation of the epigenome using WGBS data on larger cohorts will reveal inheritable and modifiable epigenetic information regarding PE that has yet been explained by traditional sequencing.

## Conclusion

Pre-eclampsia has a complex heritable genetic component that is not completely understood. Herein, we examine the methylome of 13 individuals, in 2 multiplex families and present evidence for the involvement of DMCpGs with small-effect sizes having a cumulative effect. Our gene-based analyses demonstrated that while individual dinucleotides did not reveal statistically significant results the known candidate genes, *COL4A1, SLC2A4, PER3, FLT1, GPI, LCT, DDAH1, TGFB3, DLX5*, and *LRP1B* are differentially methylated in PE cases compared to controls. Our analysis of DMRs associated with PE also identified differential methylation of three novel candidate genes, *SORD, DGKI*, and *ICA1*, which have biological functions that can be plausibly related to PE, and perhaps cardiovascular phenotypes in general. We propose that further family based genome-wide methylome analyses will provide further insights into the epigenetic landscape of PE, potentially allowing for the linking of environmental and genetic factors contributing to PE. As such, further methylomic studies are indicated to provide a better biological understanding of the disorder, which may contribute to the development of future prevention methods, diagnostic tools, and treatments.

## Author Contributions

AA conducted the bioinformatics and statistical analyses of the methylation data, assisted with the interpretation of results and wrote the first draft of the manuscript. PM provided statistical expertise and was involved in data analysis and interpretation of results. SB was responsible for the Australian sample collection and provided clinical expertise and contributed to interpretation of the findings. EM conceived and supervised the study. All co-authors reviewed and made contributions to the final manuscript.

## Conflict of Interest Statement

The authors declare that the research was conducted in the absence of any commercial or financial relationships that could be construed as a potential conflict of interest.

## Footnotes

1 https://www.bioinformatics.babraham.ac.uk/projects/trim_galore/

2 https://www.bioinformatics.babraham.ac.uk/projects/fastqc/

## Supplementary Material

FIGURE S1 Family structures of two multiplex families sequenced in this study. Each tier in the pedigree represents one generation, with relationships represented by connecting lines (horizontal lines for siblings or marriages, and vertical lines for parent-child relationships). Males are represented as squares, and females as circles. Sequenced individuals are coloured red, whereas un-sequenced individuals are black. One female affected by eclampsia is represented by a fully coloured circle, those with severe PE by half-coloured circles, those unaffected are uncoloured circles with the letter N (normotensive), and those with unknown status labelled with a question mark. Strikethroughs represent deceased individuals.

FIGURE S2 Bioinformatics pipeline for methylation annotation and analysis. Two calling platforms were used, Bismark and RnBeads. A conservative approach to filtering differentially methylated regions (DMRs) was achieved by analysing the concordance between both platforms, resulting in the final datasets for (1) all samples, and (2) only female samples.

TABLE S1 Table of sequencing statistics for each individual in this study. Columns headed by GC(%) and AT(%) represent the percentage GC and AT, respectively, whereas columns headed by Q20(%) and Q30(%) represent the percentage of reads meeting the Phred quality score criteria of 20 and 30, respectively. The final row is an average value for all sequenced individuals.

TABLE S2 Table of post-filtering read statistics for each individual in this study. Columns labelled methylated and unmethylated CpGs, CHGs, and CHHs denote the number of cytosine sites containing, respectively, methylated and unmethylated residues in the three different cytosine contexts. At the bottom of the table are given two rows denoting average putative methylation sites per genome for each methylated cytosine category (sum of methylated and unmethylated cytosine sites), as well as the percentage of these sites [methylated sites / (methylated + unmethylated sites)].

TABLE S3 List of candidate genes with their chromosomal positions under human genome build GRCh37/hg19.
