# Supplementary figures and images for: Analysis of the Epigenome in Multiplex Pre-eclampsia Families Identifies SORD, DGKI, and ICA1 as Novel Candidate Risk Genes
Source: Front Genet. 2019 Mar 19;10:227. doi: 10.3389/fgene.2019.00227 (PMC6434177; doi:10.3389/fgene.2019.00227)

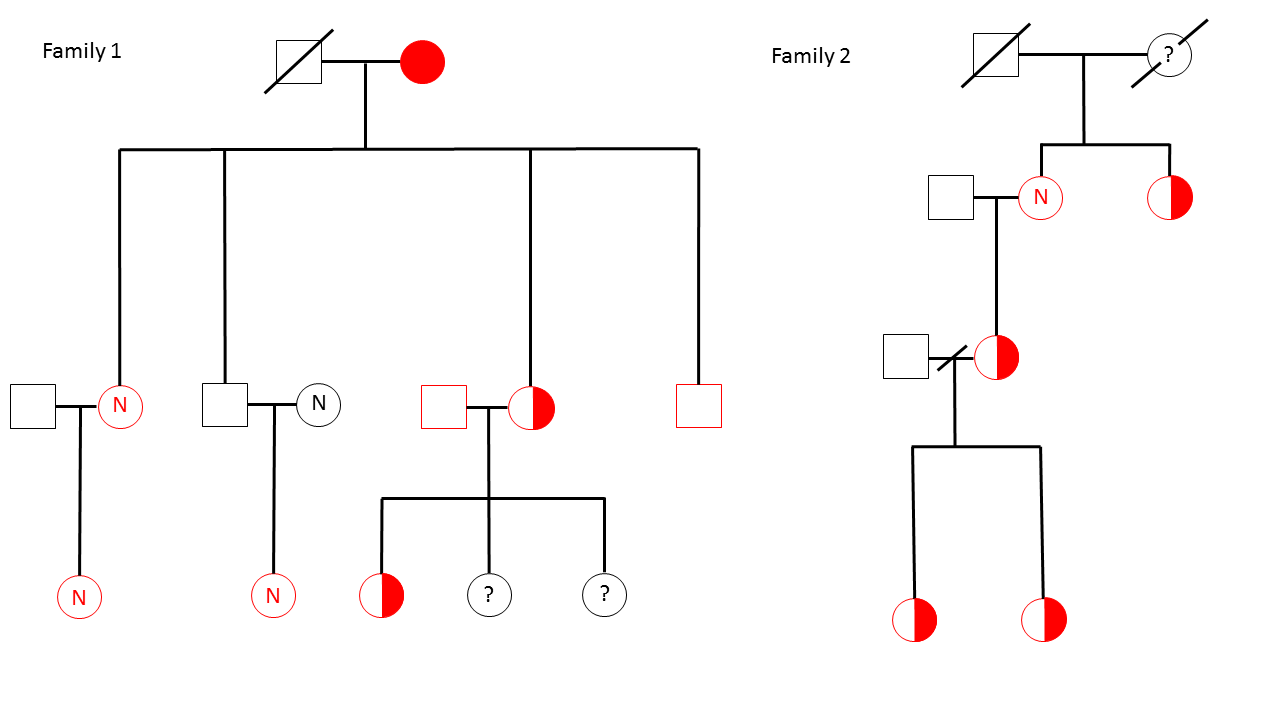

Supplement: FIGURE S1 — Family structures of two multiplex families sequenced in this study. Each tier in the pedigree represents one generation, with relationships represented by connecting lines (horizontal lines for siblings or marriages, and vertical lines for parent-child relationships). Males are represented as squares, and females as circles. Sequenced individuals are coloured red, whereas un-sequenced individuals are black. One female affected by eclampsia is represented by a fully coloured circle, those with severe PE by half-coloured circles, those unaffected are uncoloured circles with the letter N (normotensive), and those with unknown status labelled with a question mark. Strikethroughs represent deceased individuals. [file Image_1.TIF]

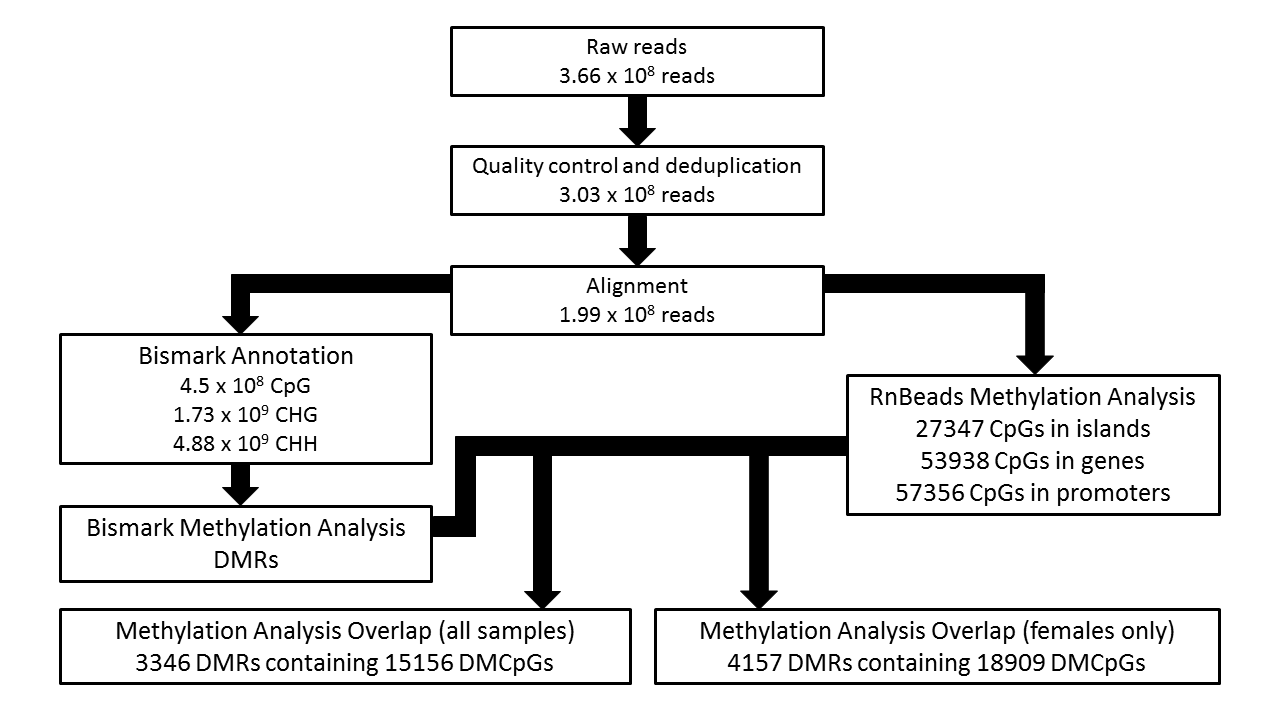

Supplement: FIGURE S2 — Bioinformatics pipeline for methylation annotation and analysis. Two calling platforms were used, Bismark and RnBeads. A conservative approach to filtering differentially methylated regions (DMRs) was achieved by analysing the concordance between both platforms, resulting in the final datasets for (1) all samples, and (2) only female samples. [file Image_2.TIF]
