# Supplementary material for: Analysis of the Epigenome in Multiplex Pre-eclampsia Families Identifies SORD, DGKI, and ICA1 as Novel Candidate Risk Genes
Source: Front Genet. 2019 Mar 19;10:227. doi: 10.3389/fgene.2019.00227 (PMC6434177; doi:10.3389/fgene.2019.00227)
Supplement: TABLE S1 — Table of sequencing statistics for each individual in this study. Columns headed by GC(%) and AT(%) represent the percentage GC and AT, respectively, whereas columns headed by Q20(%) and Q30(%) represent the percentage of reads meeting the Phred quality score criteria of 20 and 30, respectively. The final row is an average value for all sequenced individuals. [file Table_1.DOCX]

| Sample ID | Total read length (bp) | Total reads | GC(%) | AT(%) | Q20(%) | Q30(%) |
| --- | --- | --- | --- | --- | --- | --- |
| BU569 | 111,613,872,418 | 739,164,718 | 30.72 | 69.28 | 93.85 | 87.44 |
| BU572 | 114,135,218,172 | 755,862,372 | 29.98 | 70.02 | 93.56 | 86.67 |
| BU573 | 114,046,598,990 | 755,275,490 | 30 | 70 | 93.37 | 86.19 |
| BU576 | 113,734,794,258 | 753,210,558 | 30.09 | 69.91 | 93.67 | 86.75 |
| BU577 | 109,434,770,278 | 724,733,578 | 29.97 | 70.03 | 93.95 | 87.45 |
| DI470 | 108,612,299,250 | 719,286,750 | 30.49 | 69.51 | 90.92 | 81.61 |
| DI472 | 107,579,715,346 | 712,448,446 | 30.41 | 69.59 | 91.29 | 82.23 |
| DI473 | 109,767,859,064 | 726,939,464 | 29.94 | 70.06 | 91.69 | 82.68 |
| DI474 | 110,262,882,062 | 730,217,762 | 30.06 | 69.94 | 92.8 | 85.02 |
| DI475 | 109,798,970,802 | 727,145,502 | 30.65 | 69.35 | 91.69 | 82.93 |
| DI530 | 111,391,795,510 | 737,694,010 | 30.43 | 69.57 | 91.31 | 81.94 |
| DI560 | 111,573,134,732 | 738,894,932 | 31.41 | 68.59 | 90.76 | 81.22 |
| DI676 | 109,239,532,110 | 723,440,610 | 31.61 | 68.39 | 91.03 | 81.71 |
| average | 110,860,880,230 | 734,178,015 | 30 | 70 | 92 | 84 |
