# Supplementary material for: Analysis of the Epigenome in Multiplex Pre-eclampsia Families Identifies SORD, DGKI, and ICA1 as Novel Candidate Risk Genes
Source: Front Genet. 2019 Mar 19;10:227. doi: 10.3389/fgene.2019.00227 (PMC6434177; doi:10.3389/fgene.2019.00227)
Supplement: TABLE S2 — Table of post-filtering read statistics for each individual in this study. Columns labelled methylated and unmethylated CpGs, CHGs, and CHHs denote the number of cytosine sites containing, respectively, methylated and unmethylated residues in the three different cytosine contexts. At the bottom of the table are given two rows denoting average putative methylation sites per genome for each methylated cytosine category (sum of methylated and unmethylated cytosine sites), as well as the percentage of these sites [methylated sites / (methylated + unmethylated sites)]. [file Table_2.DOCX]

| Total Reads | Aligned Reads | Unaligned Reads | Ambiguously Aligned Reads | No Genomic Sequence | Duplicate Reads (removed) | Unique Reads (remaining) | Total Cs | Methylated CpGs | Unmethylated CpGs | Methylated CHGs | Unmethylated CHGs | Methylated CHHs | Unmethylated CHHs |
| --- | --- | --- | --- | --- | --- | --- | --- | --- | --- | --- | --- | --- | --- |
| 368,864,657 | 277,656,074 | 72,612,360 | 18,596,217 | 6 | 71,206,122 | 206,449,952 | 7,184,173,211 | 367,585,727 | 106,253,414 | 37,426,274 | 1,738,354,476 | 48,902,559 | 4,885,650,761 |
| 377,008,103 | 280,670,702 | 79,404,850 | 16,932,531 | 20 | 67,835,236 | 212,835,466 | 7,768,149,688 | 384,533,340 | 108,359,239 | 65,896,591 | 1,829,729,530 | 135,421,851 | 5,244,209,137 |
| 376,891,104 | 278,776,802 | 81,255,109 | 16,859,179 | 14 | 66,806,266 | 211,970,536 | 7,594,838,781 | 372,770,269 | 105,222,923 | 51,098,354 | 1,802,086,916 | 89,326,398 | 5,174,333,921 |
| 375,956,027 | 280,467,414 | 78,405,539 | 17,083,060 | 14 | 68,765,773 | 211,701,641 | 7,600,242,605 | 373,708,610 | 110,305,272 | 55,843,742 | 1,807,205,350 | 106,280,134 | 5,146,899,497 |
| 361,534,009 | 269,232,857 | 76,102,491 | 16,198,634 | 27 | 64,991,672 | 204,241,185 | 7,388,331,520 | 366,592,854 | 97,800,365 | 77,681,268 | 1,722,495,587 | 176,228,828 | 4,947,532,618 |
| 358,152,730 | 246,301,611 | 96,625,787 | 15,225,329 | 3 | 54,400,136 | 191,901,475 | 6,688,759,651 | 327,812,324 | 94,072,493 | 35,511,711 | 1,601,566,807 | 50,434,247 | 4,579,362,069 |
| 355,059,164 | 245,665,222 | 94,418,368 | 14,975,566 | 8 | 58,348,205 | 187,317,017 | 6,581,252,131 | 323,528,011 | 93,597,275 | 36,658,283 | 1,571,857,369 | 53,225,820 | 4,502,385,373 |
| 362,348,781 | 251,912,334 | 95,237,297 | 15,199,145 | 5 | 59,687,199 | 192,225,135 | 6,956,403,625 | 335,868,288 | 95,946,798 | 54,407,235 | 1,632,806,244 | 108,632,930 | 4,728,742,130 |
| 364,453,177 | 263,502,821 | 85,242,327 | 15,708,019 | 10 | 58,209,256 | 205,293,565 | 7,367,191,609 | 354,691,434 | 103,948,649 | 64,217,933 | 1,727,794,811 | 140,670,919 | 4,975,867,863 |
| 362,365,919 | 252,263,556 | 93,878,916 | 16,223,438 | 9 | 58,336,135 | 193,927,421 | 6,828,475,757 | 344,076,268 | 97,913,134 | 49,470,602 | 1,633,152,081 | 90,973,797 | 4,612,889,875 |
| 367,621,393 | 252,876,501 | 98,884,397 | 15,860,490 | 5 | 56,991,743 | 195,884,758 | 6,902,751,860 | 337,308,788 | 94,155,465 | 55,654,599 | 1,626,089,436 | 112,522,957 | 4,677,020,615 |
| 368,023,998 | 250,918,125 | 100,020,821 | 17,085,050 | 2 | 57,931,405 | 192,986,720 | 6,489,027,607 | 328,066,766 | 92,950,253 | 39,203,346 | 1,561,282,484 | 59,296,957 | 4,408,227,801 |
| 360,378,326 | 252,410,963 | 91,096,097 | 16,871,254 | 12 | 66,933,178 | 185,477,785 | 6,384,614,053 | 334,311,325 | 95,149,823 | 39,294,154 | 1,558,037,544 | 58,912,963 | 4,298,908,244 |
| average | 261,742,691 | 87,937,258 | 16,370,609 | 10 | 62,341,717 | 199,400,974 | 7,056,477,854 | 350,065,693 | 99,667,316 | 50,951,084 | 1,677,881,433 | 94,679,258 | 4,783,233,070 |
|  |  |  |  |  |  |  | average putative methylation sites per genome | 449,733,008 |  | 1,728,832,517 |  | 4,877,912,328 |  |
|  |  |  |  |  |  |  | average percentage methylation C per putative sites | 77.83855892 |  | 2.947138227 |  | 1.940979093 |  |
