# Supplementary material for: Analysis of the Epigenome in Multiplex Pre-eclampsia Families Identifies SORD, DGKI, and ICA1 as Novel Candidate Risk Genes
Source: Front Genet. 2019 Mar 19;10:227. doi: 10.3389/fgene.2019.00227 (PMC6434177; doi:10.3389/fgene.2019.00227)
Supplement: TABLE S3 — List of candidate genes with their chromosomal positions under human genome build GRCh37/hg19. [file Table_3.DOCX]

| gene | chr | start | stop | full gene name |
| --- | --- | --- | --- | --- |
| ACVR1C | chr2 | 157526767 | 157629005 | activin A receptor type IC |
| ACVR1 | chr2 | 157736449 | 157875862 | activin A receptor type I |
| ACVR2A | chr2 | 147844517 | 147930824 | activin A receptor type IIA |
| COL4A1 | chr13 | 110148963 | 110307149 | collagen type IV alpha 1 |
| COL4A2 | chr13 | 110307267 | 110513027 | collagen type IV alpha 2 |
| ERAP1 | chr5 | 96760810 | 96808100 | endoplasmic reticulum aminopeptidase 1 |
| ERAP2 | chr5 | 96875939 | 96919716 | endoplasmic reticulum aminopeptidase 2 |
| INHA | chr2 | 219572195 | 219575713 | inhibin alpha |
| INHBB | chr2 | 120346143 | 120351808 | inhibin beta B |
| LNPEP | chr5 | 96935464 | 97037515 | leucyl/cystinyl aminopeptidase |
| AVP | chr20 | 3082556 | 3084724 | arginine vasopressin |
| CYP17A1 | chr10 | 102830531 | 102837533 | cytochrome P450 family 17 subfamily A polypeptide 1 |
| FST | chr5 | 53480409 | 53487134 | follistatin |
| OXT | chr20 | 3071620 | 3072517 | oxytocin/neurophysin I prepropeptide |
| SHH | chr7 | 155799986 | 155812273 | sonic hedgehog |
| SLC2A4 | chr17 | 7281667 | 7288257 | solute carrier family 2 (facilitated glucose transporter) member 4 |
| SMAD7 | chr18 | 48919853 | 48950711 | SMAD family member 7 |
| XIAP | chrX | 123859812 | 123913976 | X linked inhibitor of apoptosis E3 ubiquitin protein ligase |
| CD72 | chr9 | 35609533 | 35619542 | CD72 molecule |
| DBP | chr19 | 48630030 | 48637438 | D site of albumin promoter (albumin D box) binding protein |
| DPP7 | chr9 | 137110542 | 137114718 | dipeptidyl peptidase 7 |
| HS3ST2 | chr16 | 22814177 | 22916338 | heparan sulfate (glucosamine) 3 O sulfotransferase 2 |
| PDK4 | chr7 | 95583499 | 95596491 | pyruvate dehydrogenase kinase isozyme 4 |
| PER3 | chr1 | 7784703 | 7845177 | period circadian clock 3 |
| SLC2A6 | chr9 | 133471095 | 133479137 | solute carrier family 2 (facilitated glucose transporter) member 6 |
| TNFRSF14 | chr1 | 2556366 | 2563829 | tumor necrosis factor receptor superfamily member 14 |
| CDH1 | chr16 | 68737225 | 68835548 | cadherin 1 type 1 E cadherin |
| EDN1 | chr6 | 12290363 | 12297194 | endothelin 1 |
| ENG | chr9 | 127815017 | 127854636 | endoglin |
| FLT1 | chr13 | 28300344 | 28495095 | fms related tyrosine kinase 1 |
| IL10 | chr1 | 206767602 | 206772494 | interleukin 10 |
| INS | chr11 | 2159779 | 2161341 | Insulin |
| KDR | chr4 | 55078477 | 55125589 | kinase insert domain receptor |
| MMP2 | chr16 | 55478971 | 55506691 | matrix metallopeptidase 2 |
| MMP9 | chr20 | 46008908 | 46016561 | matrix metallopeptidase 9 |
| NOS2 | chr17 | 27756766 | 27800499 | nitric oxide synthase 2 |
| NOS3 | chr7 | 150990995 | 151014588 | nitric oxide synthase 3 |
| PTGS2 | chr1 | 186671791 | 186680427 | prostaglandin endoperoxide synthase 2 |
| TNF | chr6 | 31575567 | 31578336 | tumor necrosis factor |
| ACP5 | chr19 | 11685475 | 11689801 | acid phosphatase 5 tartrate resistant |
| ADIPOR2 | chr12 | 1800247 | 1897845 | adiponectin receptor 2 |
| ALDOA | chr16 | 30076994 | 30081741 | aldolase A fructose bisphosphate |
| ANKFY1 | chr17 | 4066665 | 4167142 | ankyrin repeat and FYVE domain containing 1 |
| BMF | chr15 | 40380091 | 40401085 | Bcl2 modifying factor |
| CEP152 | chr15 | 49030135 | 49103343 | centrosomal protein 152kDa |
| CYGB | chr17 | 74523430 | 74533987 | cytoglobin |
| FAM21A | chr10 | 51827684 | 51893269 | family with sequence similarity 21 member A |
| GPI | chr19 | 34856032 | 34893318 | glucose 6 phosphate isomerase |
| GTF3C4 | chr9 | 135545728 | 135565470 | general transcription factor IIIC polypeptide 4 |
| ISPD | chr7 | 16127152 | 16460947 | isoprenoid synthase domain containing |
| LMAN1 | chr18 | 56995056 | 57026508 | lectin mannose binding 1 |
| LY9 | chr1 | 160765864 | 160798045 | lymphocyte antigen 9 |
| P2RY11 | chr19 | 10222197 | 10226064 | purinergic receptor P2Y G protein coupled 11 |
| RENBP | chrX | 153200722 | 153210232 | renin binding protein |
| SLC27A2 | chr11 | 124933013 | 124960412 | solute carrier family 37 |
| TEF | chr22 | 41777933 | 41795332 | thyrotrophic embryonic factor |
| XPO5 | chr6 | 43490068 | 43543812 | exportin 5 |
| ZDHHC9 | chrX | 128937264 | 128977910 | zinc finger DHHC type containing 9 |
| AGT | chr1 | 230702523 | 230714297 | angiotensinogen |
| IFNG | chr12 | 68154768 | 68159747 | interferon gamma |
| IL6 | chr7 | 22725884 | 22732002 | interleukin 6 |
| INHBA | chr7 | 41685114 | 41703108 | inhibin beta A |
| SERPINE1 | chr7 | 101127089 | 101139266 | serpin peptidase inhibitor clade E |
| TGFB1 | chr19 | 41330323 | 41353911 | transforming growth factor beta 1 |
| VEGFA | chr6 | 43770707 | 43784949 | vascular endothelial growth factor A |
| STOX2 | chr4 | 183905356 | 184023526 | storkhead box 2 |
| QRFP | chr9 | 130892702 | 130896812 | Pyroglutamylated RFamide Peptide |
| QRFPR | chr4 | 121329314 | 121381059 | Pyroglutamylated RFamide Peptide Receptor |
| STOX1 | chr10 | 68827541 | 68895432 | storkhead box 1 |
| LCT | chr2 | 136545415 | 136594750 | lactase |
| GCA | chr2 | 163200583 | 163219148 | grancalcin EF-hand calcium binding protein |
| LRP1B | chr2 | 140988996 | 142889270 | low density lipoprotein receptor-related protein 1B |
| DDAH1 | chr1 | 85784168 | 85930889 | dimethylarginine dimethylaminohydrolase 1 |
| TGFB3 | chr14 | 76424442 | 76448092 | transforming growth factor beta 3 |
| DLX5 | chr7 | 96649702 | 96654143 | distal-less homeobox 5 |
| ACE | chr17 | 61554422 | 61575741 | angiotensin I converting enzyme |
| CTLA4 | chr2 | 204732511 | 204738683 | Homo sapiens cytotoxic T lymphocyte associated protein 4 |
| F2 | chr11 | 46740743 | 46761056 | coagulation factor II thrombin |
| FV | chr1 | 169481192 | 169555769 | coagulation factor V |
| LPL | chr8 | 19796582 | 19824770 | lipoprotein lipase |
